# Retracted: 5-Aminolevulinic Acid-Based Photodynamic Therapy Pretreatment Mitigates Ultraviolet A-Induced Oxidative Photodamage

**DOI:** 10.1155/2020/2769472

**Published:** 2020-08-29

**Authors:** Oxidative Medicine and Cellular Longevity

*Oxidative Medicine and Cellular Longevity* has retracted the article titled “5-Aminolevulinic Acid-Based Photodynamic Therapy Pretreatment Mitigates Ultraviolet A-Induced Oxidative Photodamage” [1]. After concerns were raised on PubPeer the article was found to contain duplicated images, where part of the second image (ALA-PDT-UVA) in Figure 1c is the same as the third image (ALA-PDT-UVA) in Figure 2a.

The authors stated that this error was due to negligence in preparation of the manuscript. They apologized for not noticing it and clarified that the author who edited Figure 1 misunderstood the label of the image file and used the wrong image and that they missed noticing this in the other versions. They said the error does not affect the experimental statistics or the final conclusion of the study and provided a replacement for the duplicated panel in Figure 1c.

However, the Editorial Board recommended retraction. The authors agree to retraction.
